# Functional interleukin-4 releasing microparticles impact THP-1 differentiated macrophage phenotype

**DOI:** 10.3389/fbioe.2024.1496111

**Published:** 2024-11-05

**Authors:** I-Ning Lee, Jasmine Z. Stening, Felicity R. A. J. Rose, Lisa J. White

**Affiliations:** School of Pharmacy, Nottingham Biodiscovery Institute, University of Nottingham, University Park, Nottingham, United Kingdom

**Keywords:** drug delivery, microparticles, IL-4, release kinetics, macrophages, THP-1, controlled release

## Abstract

**Introduction:**

Macrophage cell therapies offer potential treatment in inflammatory diseases due to their ability to mobilize and stimulate their environment. However, successful treatment requires a pro-regenerative macrophage phenotype to be retained *in vivo*. Polymeric microparticles may provide a potential route to direct and sustain macrophage phenotype. Interleukin-4 (IL-4) is the most commonly used cytokine for *in vitro* modulation towards M2a macrophage phenotype. We designed IL-4 encapsulated microparticles to investigate the impact of drug release kinetics and developed a robust human peripheral blood monocyte cell (THP-1) *in vitro* assay to assess functional IL-4 release upon macrophage phenotype.

**Methods:**

IL-4 was encapsulated with human serum albumin (HSA) in microparticles fabricated from a blend of PLGA and a PLGA-PEG-PLGA triblock copolymer. Functional release of IL-4 and HSA over different time periods was measured using ELISAs. THP-1 differentiated macrophages were cultured either in direct contact with microparticles or indirectly through transwells. The immunomodulatory impact of microparticles on THP-1 cells were measured using ELISA and qPCR.

**Results and Discussion:**

IL-4 release kinetics fit with the first-order release kinetics model, indicating concentration dependent release. IL-4/HSA encapsulated microparticles modulated THP-1 differentiated macrophages towards pro-immunoregulatory subgroups. This strategy provides a novel approach in drug carrier development for *in vitro* assessments of macrophage phenotype to inform development of targeted therapies for inflammation and immune modulation.

## Graphical Abstract

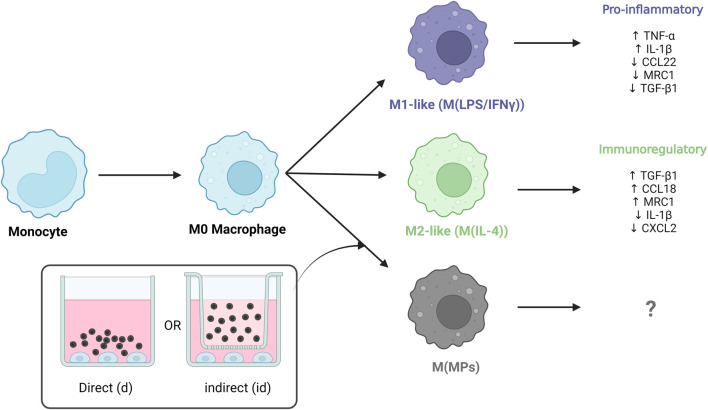

## 1 Introduction

Macrophages are derived from bone marrow progenitor cells and either enter circulation to form monocytes or infiltrate into tissues to become resident macrophages (Das et al., 2015). Circulating monocytes are recruited to the injury site by pro-inflammatory cytokines such as TNFα and IL-1, where they differentiate into macrophages (Alvarez et al., 2016). Once differentiated these cells become key mediators of inflammation and the removal of foreign bodies though the means of phagocytosis (Mamilos et al., 2023). Pro-inflammatory macrophages are often referred to as “M1” macrophages as they are stimulated by cytokines associated with type 1 helper (Th1) cells (Risser et al., 2023). Efficient accumulation of pro-inflammatory macrophages at the early stage of injury contributes to the initiation of healing whilst at later stages, the shift to pro-immunoregulatory phenotypes promotes stabilization and tissue maturation (Spiller and Koh, 2017). Pro-immunoregulatory macrophages have been referred to as “M2” macrophages as anti-inflammatory, immunosuppressive behaviours can be stimulated by Th2 cytokines.

Throughout the tissue healing process, macrophage phenotype changes in response to microenvironmental cues, and macrophages exist in an evolving continuum between the proinflammatory and proregulatory states (Martinez and Gordon, 2014; Yunna et al., 2020). Macrophages expressing M2 markers may have diverse and opposing functions; multiple M2-like phenotypes have been identified and categorised as M2a, M2b, M2c and M2d (Strizova et al., 2023).

Macrophage cell therapies have gained considerable interest in the treatment of numerous inflammatory diseases due to macrophage ability to mobilize and stimulate their environment (Na et al., 2023). However, studies of *ex-vivo* activated macrophages have not provided consistent results which may be due to loss of desired macrophage phenotype, required to promote regeneration. Thus, there is a need for methods to maintain macrophage phenotype *in vivo*. Biomaterials strategies to promote specific macrophage phenotypes have had some success with cytokine incorporated vascular grafts (Tan et al., 2019) and polymer films (Ziemba et al., 2019) modulating macrophage phenotype and suppressing local inflammation. Microparticles provide a biomaterial strategy that can deliver cytokines to direct macrophage phenotype in an injectable format that can be co-administered with macrophages. Microparticles fabricated with synthetic degradable polymers such as poly (lactic-co-glycolic acid) (PLGA), poly (lactic acid) (PLA), and polycaprolactone (PCL) have been widely used in drug delivery for their ability to prevent drug or cytokine degradation (White et al., 2013; Rambhia and Ma, 2015). Drugs delivered through microparticle systems increase their half-life *in vivo* and ensure localised delivery while providing relatively low yet sustained dosage in a prolonged period (da Silva et al., 2023).

Recently, Risser et al. demonstrated beneficial impacts on angiogenesis in a mouse hindlimb ischemic model with co-administered macrophages and IL-4 releasing microparticles (Risser et al., 2023), demonstrating the potential of microparticles to direct macrophage phenotype. However, more in-depth knowledge is needed regarding the impact of controlled release of cytokines on macrophage phenotype *in vitro*, prior to *in vivo* applications. It is imperative to develop *in vitro* assessments of sustained macrophage phenotype in order to reduce reliance on *in vivo* models (Hubrecht and Carter, 2019). This study aims to fill these gaps in knowledge to advance understanding of how tailored rates of release of cytokines can be used to direct and sustain macrophage phenotype to improve efficacy of macrophage therapies.

We have previously developed robust controlled release microparticle systems to improve localised delivery of drugs and cytokines *in vivo* (Kirby et al., 2011; White et al., 2013). However, our previous microparticle systems were large (50–100 μm) so we first designed microparticles to meet the constraints of co-administration by injection or infusion and to be larger than 10 μm, to limit phagocytosis by macrophages. As IL-4 stimulated macrophages are the most commonly studied M2 subtype *in vitro* (Risser et al., 2023), and IL-4 has been shown to modulate macrophage phenotype from a classically activated pro-inflammatory phenotype to an alternatively activated pro-immunoregulatory phenotype, M2a *in vivo* (Arora et al., 2018; Mantovani et al., 2014; Sica and Mantovani, 2012) we encapsulated IL-4 and explored the drug release kinetics. Whilst there has been a wealth of *in vitro* studies conducted using chemical stimuli and cytokines to polarize macrophages and examine macrophage and cell interactions (Sung et al., 2023), there are currently no well-developed explorations of functional *in vitro* release of cytokines from microparticles and consequent impact on macrophage phenotype. Thus, in this study, we developed an efficient *in vitro* model to assess the effect of IL-4 released from microparticles on macrophage phenotype.

## 2 Materials and methods

### 2.1 Synthesis of poly (lactic-coglycolic acid)-poly (ethylene glycol)-poly (lactic-coglycolic acid) triblock copolymer (TB)

A mixture of poly (ethylene glycol) (5.5 g, 1,500 Da, Sigma-Aldrich, United Kingdom), glycolide (3.08 g, Fisher Scientific Ltd., United Kingdom) and D,L-Lactide (9.57 g, Fisher Scientific Ltd., United Kingdom) was dried under vacuum for 1 h at 100°C. The temperature was then increased to 150°C followed by the addition of Tin (II) 2-ethylhexanoate (29 μL, Sigma-Aldrich, United Kingdom) in a dropwise manner. The reaction was carried out for 5 h under dry nitrogen. After which the reaction mixture was cooled to room temperature and subsequently poured into 250 mL of dichloromethane (DCM) until complete dissolution. The solution was then purified by pouring into 250 mL of ice-cold diethyl ether. The precipitation was collected and the purification process was repeated for a total number of 3 times. The purified polymer was then collected and residual solvent evaporated under vacuum overnight. The product was characterised using GPC (Shimadzu, Japan) and NMR (Bruker 400 MHz). ^1^H NMR (400 MHz, CDCl_3_) δ 5.27 (s, 1H), 5.23–5.07 (m, 5H), 4.89–4.60 (m, 3H), 4.36–4.20 (m, 1H), 3.60 (s, 20H), 3.44 (q, *J* = 7.0 Hz, 5H), 1.65–1.40 (m, 15H), 1.16 (t, *J* = 7.0 Hz, 8H). The resultant triblock co-polymer had Mw 6,090 Da, Mn 4,940 Da, and PDI 1.23.

### 2.2 Microparticle fabrication and characterization

Microparticles were fabricated using a double emulsion method as previously described (White et al., 2013). In brief, a mixture of PLGA (85:15, 55 kDa, IV 0.47 dL/g, Evonik Industries AG) with various amounts of TB at a total mass of 1 g was dissolved in 5 mL of DCM. An aqueous solution of human serum albumin (HSA, 100 mg mL^−1^) or a mixture of HSA (95 mg mL^−1^, Sigma-Aldrich, United Kingdom) and IL-4 (5 mg mL^−1^, Peprotech, United Kingdom), was prepared. The use of HSA has previously been shown to stabilise cytokines during the encapsulation process and enhance overall encapsulation efficiency (Pean et al., 1998; White et al., 2013). One hundred microlitres of protein solution was added to the polymer solution and homogenised using a Silverson L5M homogeniser (Silverson Machines, United Kingdom) at 4,000 rpm for 2 min to form the primary water-in-oil emulsion. The emulsion was then poured into 200 mL of 0.3% (w/v) poly (vinyl alcohol)_(aq)_ (13,000–23,000 Da, 87%–89% hydrolysed, Sigma-Aldrich, United Kingdom) and homogenised at 9,000 rpm for 2 min. The product was then stirred at 300 rpm on a stirrer plate for a minimum of 4 h to facilitate solvent evaporation. The microparticles were then washed with copious amounts of water followed by centrifugation at 2,100 × g after each wash. The final product was then lyophilised using a CoolSafe 4–15 L freeze dryer (LaboGene, Denmark). The size distribution of microparticles was determined using laser diffraction particle size analysis (LA-960, HORIBA Scientific Ltd.). Qualitative images of the microparticles were obtained by sputter coating with gold at 5 nm thickness using Balzers SCD 030 sputter coater (Balzers, Liechtenstein) and imaging using scanning electron microscopy (FEI XL30, Hillsboro, OR).

### 2.3 Protein encapsulation efficiency

Ten milligrams of microparticles were dissolved in 750 µL of dimethyl sulfoxide, followed by addition of 2.15 mL of 0.02% (w/v) sodium dodecyl sulfate in 200 mM sodium hydroxide solution for 1 h. The concentration of protein encapsulated was assessed using a micro BCA™ protein assay (Fisher Scientific Ltd., United Kingdom) according to manufacturer’s instructions. Standard curves were obtained using HSA in concentrations ranging from 30 to 2 μg mL^−1^.

### 2.4 Protein release from microparticles and mathematical modelling of functional release

Twenty-five milligrams of microparticles were suspended in 1.5 mL of complete culture media, described in Section 2.6. The samples were incubated at 37°C on a rocker continuously at 60 rpm. At appropriate time intervals, the samples were briefly centrifuged, supernatant was collected and replaced with fresh media. Functional release of IL-4 and HSA were measured using ELISA kits (DY204 and DY1455, R&D Systems, United Kingdom) according to manufacturer’s instructions.

Four commonly used drug release kinetic models were selected to analyse the controlled release kinetics: zero order, first order, Higuchi and Ritger-Peppas (Peppas and Narasimhan, 2014; Li et al., 2021; Trucillo, 2022). The correlation coefficient values, R^2^, were used to select the model that best fitted the release profile.

### 2.5 Human peripheral blood monocyte culture

Human peripheral blood monocyte cells (THP-1, ATCC^®^ TIB-202™, ATCC, United Kingdom) were cultured as previously described and used within 20 population doublings upon receipt (Huleihel et al., 2017). Cultures were expanded under an atmosphere of 5% CO_2_ at 37°C in Roswell Park Memorial Institute 1,640 medium (RPMI-1640, 52400025, Thermo Fisher Scientific, United Kingdom) supplemented with 10% fetal bovine serum (Sigma-Aldrich, United Kingdom) and ß-mercaptoethanol (Fisher Scientific Ltd., United Kingdom) to a final concentration of 0.05 mM.

### 2.6 THP-1 differentiation into functional macrophages and cytokine induced polarisation

To differentiate THP-1 into functional macrophages, THP-1 cells were plated at a density of 3 × 10^5^ cells cm^−2^ in complete RPMI-1640 medium (as described above) supplemented with 300 nM of phorbol 12-myristate 13-acetate (PMA, Sigma-Aldrich, United Kingdom) for 24 h. The culture was then replaced with complete RPMI-1640 medium, with absence of PMA, for 48 h.

Functional macrophages were polarised into pro-inflammatory-like or pro-immunoregulatory-like phenotypes by direct treatment of cytokines. Pro-inflammatory-like phenotype was induced in complete RPMI-1640 medium supplemented with 100 ng mL^−1^ of lipopolysaccharide (LPS, Sigma-Aldrich, United Kingdom) and 20 ng mL^−1^ interferon-γ (IFN-γ, Peprotech, United Kingdom) for up to 72 h. Pro-immunoregulatory-like phenotype was induced in complete RPMI-1640 medium supplemented with 20 ng mL^−1^ IL-4 for up to 72 h.

### 2.7 IL-4 encapsulated microparticles to induce pro-immunoregulatory activation of THP-1 derived functional macrophages

In a 1.5 mL microcentrifuge tube, microparticles of dosage adjusted to 20 ng mL^−1^ were suspended in complete RPMI-1640 medium. In a 24-well plate, suspensions of microparticles were co-cultured either in direct contact or indirectly with functional macrophages through transwell plate inserts (membrane size of 1 μm, 83.3932.101, Sarstedt, Germany) to result in IL-4 dosage equivalent to exogenous administration of IL-4. At each time interval, media was removed and stored at −80°C until cytokine secretion analysis was undertaken withELISAs. Separately, cells were lysed at each time interval with 150 µL of Buffer RLT Plus provided with RNeasy Plus Mini Kit (74134, Qiagen, United States) and stored at −80°C until gene expression analysis was undertaken.

### 2.8 Analysis of cytokine secretion and gene expression of cytokine polarised macrophages

Cytokine secretion of macrophages cultured as described above (Section 2.7) was determined using ELISA. The pro-inflammatory-like phenotype was determined bydetection of functional human TNF-α (DY210, R&D Systems, United Kingdom) according to manufacturer’s instructions. The pro-immunomodulatory-like phenotype was indicated by measuring functional human TGF-β1 (DY240, R&D Systems, United Kingdom) and CCL18 (DY394, R&D Systems, United Kingdom), according to manufacturer’s instructions.

Gene expression profiles of macrophages cultured as described above (Section 2.7) were determined using RT-qPCR. Total RNA was isolated using RNeasy plus mini kit (74134, Qiagen, United States of America) according to manufacturer’s instructions. Nucleic acid concentrations were measured using a Nanodrop (Labtech, United Kingdom). Complementary deoxyribonucleic acid (cDNA) was then synthesized immediately with 45 ng μL^−1^ of purified RNA using High-Capacity cDNA Reverse Transcription Kit (4368814, Applied Biosystems, United Kingdom) with RNase Inhibitor (10615995, Applied Biosystems, United Kingdom) according to manufacturer’s instructions.

The synthesized cDNA samples were diluted 1:10 with nuclease-free water. To each well of a 384-well qPCR reaction plate, 5 µL of PowerUp SYBR Green Master Mix (15350929, Applied Biosystems, United Kingdom), 0.5 µL of selected primers, 2.5 µL of nuclease-free water and 2 µL of cDNA were added to yield in a 10 µL reaction volume. The reaction plate was sealed and qPCR was conducted using a Bio-Rad CFX384 Touch Real-Time PCR Detection System for 40 cycles at 60 °C. Primers used for qPCR were GAPDH (NM_002046.7, QT00079247, Qiagen, United States), CXCL2 (NM_002089.4, QT00013104, Qiagen, United States), MRC1 (NM_002438, QT00012810, Qiagen, United States), CCL22 (NM_002990.5, QT00089817, Qiagen, United States of America), IL-1β (NM_000576.3, Forward (5′-3′): CTA​AAC​AGA​TGA​AGT​GCT​CC; Reverse (5′-3′): GGT​CAT​TCT​CCT​GGA​AGG, Sigma-Aldrich, United Kingdom).

Relative expression was normalised to GAPDH and expression in functional macrophages using ΔΔCt method.

### 2.9 Statistical analysis

Statistical analyses were performed using one-way ANOVA using Origin software. Where indicated n represents the number of independent experiments per condition performed in triplicate and N represents the batches of microparticles fabricated. Statistical analysis of two batches of IL-4/HSA microparticle cumulative release best-fit kinetics were performed with a two-sample t-test using Origin software.

## 3 Results

### 3.1 Microparticle formulations, size and morphology

**FIGURE 1:**
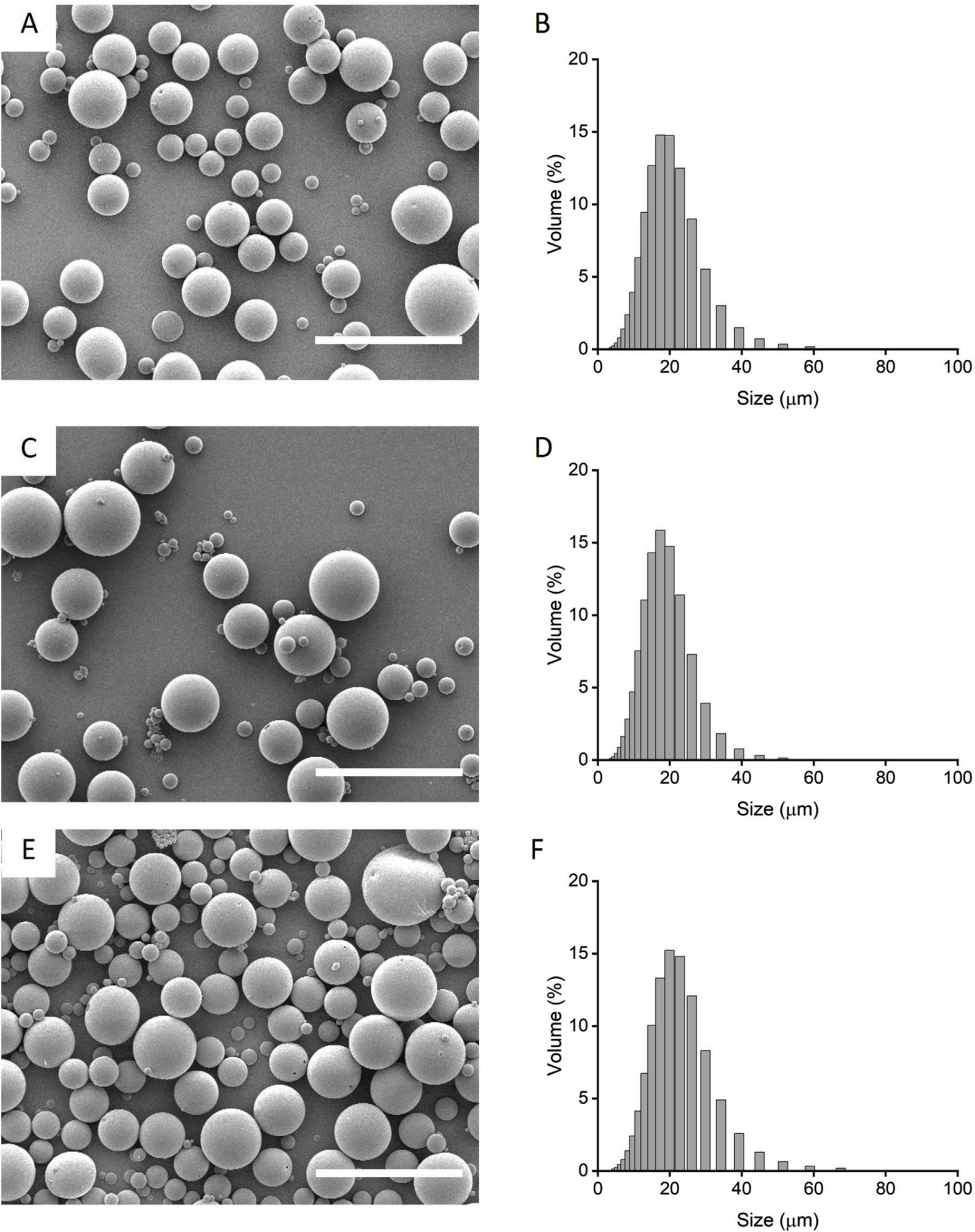
Qualitative scanning electron micrographs **(A, C, E)** and quantitative laser diffraction size distribution **(B, D, F)** of microparticles fabricated with PLGA85:15 incorporated with 40% TB **(A, B)** with absence of protein, i.e., blank; **(C, D)** encapsulated with HSA alone and, **(E, F)** encapsulated with IL-4/HSA. Scale bars represent 50 µm.

Preliminary formulation screening of microparticle release profiles were fabricated from PLGA85:15 with 0%, 20% and 40% TB, encapsulated with lysozyme/HSA at 1:9 ratio was used as model protein (Supplementary Figure S1). PLGA85:15 with 40% TB showed a sustained, linear release profile and this was chosen to fabricate microparticles encapsulated with HSA and IL-4. The morphology and size distribution of the microparticles were characterized qualitatively by scanning electron microscopy and quantitatively by laser-diffraction size distribution (Figure 1). Microparticles displayed an overall smooth and non-porous surface morphology with a bell-curved distribution of microparticle diameter. Microparticles with IL-4 and HSA (IL-4/HSA), or HSA alone did not exhibit apparent differences in morphology or size distribution. The mean diameter of microparticles without protein (i.e., blank), encapsulated with HSA and encapsulated with IL-4/HSA was 18.01 ± 7.01 µm, 16.79 ± 6.06 µm and 20.23 ± 7.84 µm (mean ± s. d.), respectively. Microparticles encapsulated with IL-4/HSA yielded a mean efficiency of 68.43% ± 6.69% encapsulation.

### 3.2 Protein release kinetics of microparticles

**FIGURE 2:**
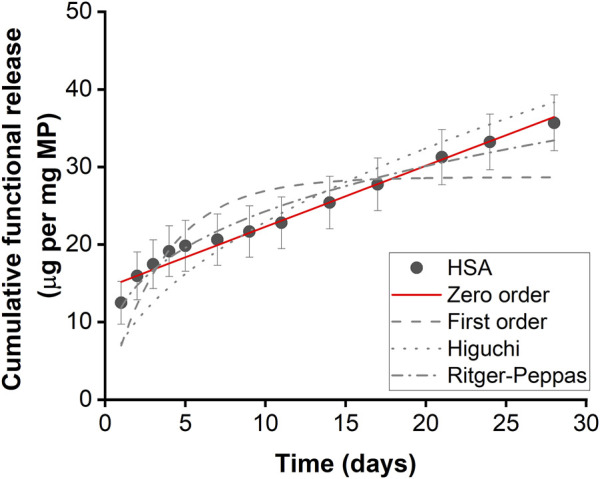
Functional HSA released from HSA encapsulated microparticles. Functional release kinetics were fitted with zero order dynamics, first order dynamics, the Higuchi dynamics, and Ritger-Peppas dynamics. Error bars represent ± cumulative standard deviation; n = 3.

Microparticles encapsulated with HSA exhibited controlled linear release kinetics (Figure 2; Table 1). Microparticles encapsulated with HSA alone had a burst release of 12.49 µg per mg of particles within the first 24 h followed by sustained release for up to 28 days. HSA encapsulated microparticles displayed a release profile that best-fitted zero-order kinetics suggesting that this formulation has the potential to provide linear drug release. Therefore, this formulation was used to fabricate IL-4 incorporated microparticles.

**TABLE 1:** Kinetics parameters of protein released from microparticles for 28 days.

Model	Equation	Parameters	HSA-alone	IL-4 N1	IL-4 N2	HSA N1	HSA N2
Zero order	Q = K_0_t + Q_0_	K_0_	0.787	0.005	0.004	0.474	0.341
		Q_0_	14.417	0.102	0.237	19.452	26.381
		R^2^	**0.969**	0.627	0.418	0.741	0.729
First order	Q = 1 – *e* ^−K^_1_^t^	K_1_	0.237	0.574	0.794	0.825	0.963
		R^2^	0.649	**0.992**	**0.971**	0.837	0.717
Higuchi	Q = K_H_t^1/2^	K_H_	7.175	0.052	0.082	7.700	8.649
		R^2^	0.751	0.229	−2.605	−1.678	−6.468
Ritger-Peppas	Q = Kt^n^	K	11.689	0.096	0.210	17.741	23.930
		R^2^	0.957	0.877	0.754	**0.950**	**0.941**

Q is the cumulative amount of drug released at time t; K_0_, K_1_, K_H_, and K are regarded as kinetic constant for zero-order release, first-order release, Higuchi, and Ritger-Peppas, respectively. And n is the diffusional exponent denoting the drug transport mechanism.

**FIGURE 3:**
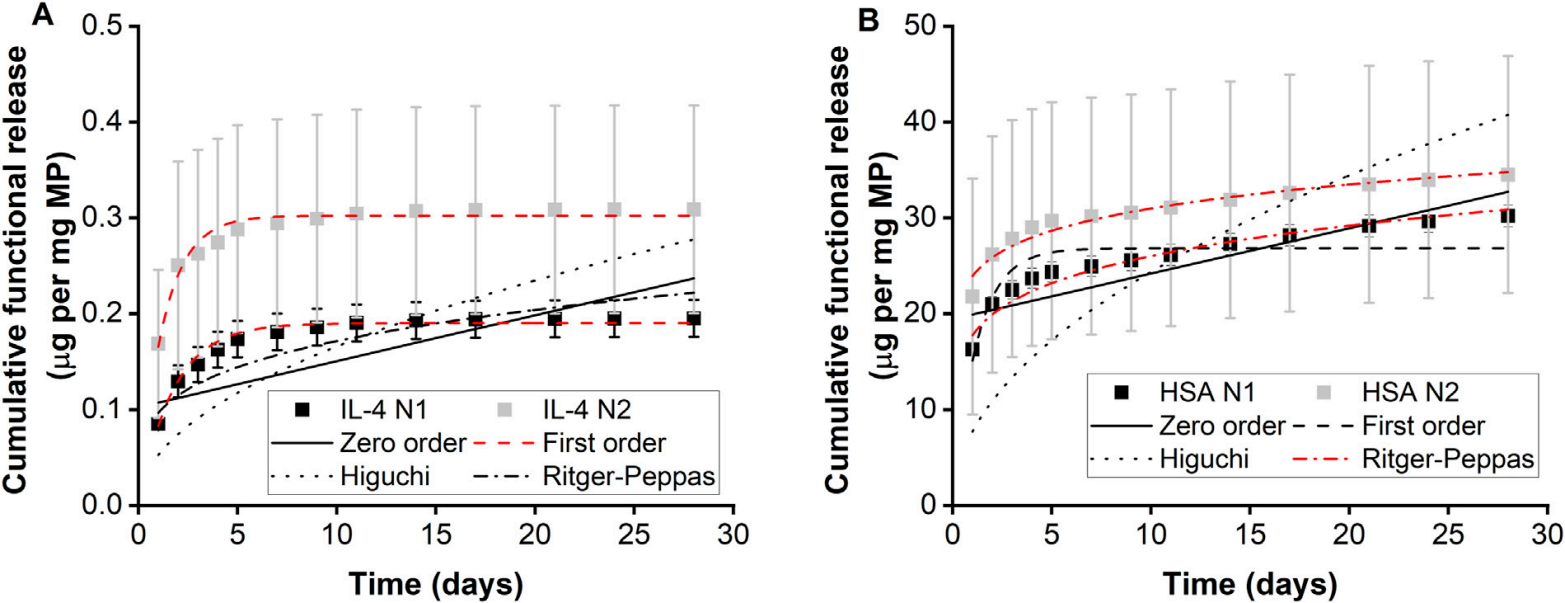
Functional protein release of microparticles measured using ELISA. Cumulative release of **(A)** IL-4 and **(B)** HSA from IL-4/HSA encapsulated microparticles, n = 3. Error bars represent ± cumulative standard deviation.

Two separate batches of IL-4/HSA microparticles were fabricated and release kinetics were examined (N1 and N2) (Table 1; Figure 3A). The functional release of IL-4 over 28 days fitted best with first order kinetics. Microparticles fabricated with IL-4 (N1) displayed an initial burst release of 85.13 ng per mg of particles within the first 24 h followed by a sustained total release of 95.81 ng of IL-4 per mg of particles over the course of 2–7 days (Figure 3A). Microparticles fabricated with IL-4 (N2) displayed an initial burst release of 168.73 ng per mg of particles within 24 h followed by a sustained total release of 125.6 ng of IL-4 per mg of particles over the course of 2–7 days. It is worth noting functional IL-4 release kinetics over both 7 days and 28 days were best fitted with first order kinetics (Supplementary Table S1; Supplementary Figure S2A).

Functional release of HSA from IL-4/HSA microparticles displayed similar release kinetics as IL-4 for both N1 and N2 batches (Table 1; Figure 3B). However, HSA release over 28 days fitted best with the Ritger-Peppas model. Although HSA release from IL-4/HSA microparticles for 7 days were best-fitted with first order kinetics (Supplementary Table S1; Supplementary Figure S2B), microparticles had continued to release HSA for 28 days.

### 3.3 Modulation of THP-1 as an *in vitro* model for cytokine release from microparticles

THP-1 differentiated functional macrophages were treated exogenously with LPS and IFN-γ for up to 72 h for modulation towards a pro-inflammatory-like phenotype (M(LPS/IFN-γ)) or treated with IL-4 for up to 72 h to modulate towards a pro-immunomodulatory-like phenotype (M(IL-4)). Microparticles with no protein (blank) and microparticles with HSA alone were used as controls. Microparticles formulations containing no protein (blank), HSA, and IL-4/HSA were cultured in direct contact (d) with macrophages or indirectly (id) through transwells plate inserts.

**FIGURE 4:**
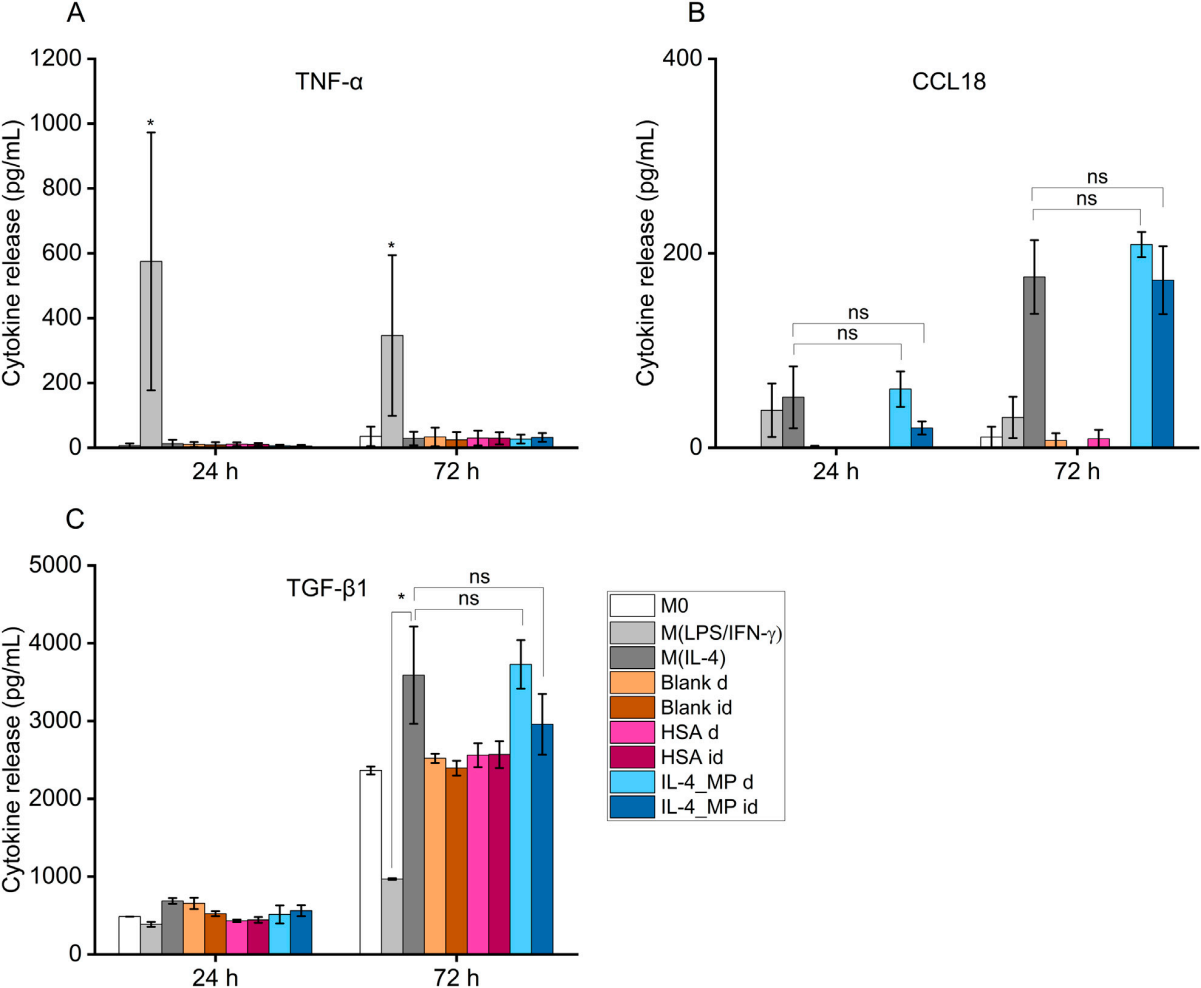
Cytokine secretion of THP-1 differentiated functional macrophages (M0), exogenously treated with pro-inflammatory cytokines, LPS and IFN-γ (M(LPS/IFN-γ)), or pro-immunoregulatory cytokines, IL-4 (M(IL-4)) in comparison to IL-4/HSA encapsulated microparticles (IL-4_MP), blank particles and HSA encapsulated microparticles (HSA) co-cultured directly (d) or indirectly (id) with cells. Quantitative expression of **(A)** TNF-α, **(B)** CCL18 and **(C)** TGF-β1 using ELISA for 24 h and 72 h post-exogenous cytokine treatments. N = 2, n = 2. Error bars represent ±standard error; significance determined using one-way ANOVA with tukey *post hoc* test, *: *p* < 0.05, significant; ns: not significant.

Cells exogenously treated with pro-inflammatory factors (M(LPS/IFN-γ)) displayed an increased secretion in TNF-α at both 24 h and 72 h (Figure 4A). In contrast, cells treated with pro-immunomodulatory factors (M(IL-4)) and blank, HSA and IL-4/HSA microparticles secreted significantly lower levels of TNF-α.

IL-4/HSA microparticles co-cultured directly or indirectly with cells exhibited increased levels of CCL18 and TGF-β1 secretion, similar to M(IL-4) polarised candidates (Figures 4B, C). M(LPS/IFN-γ) polarised candidates displayed a reduction of TGF-β1 secretion upon 72 h polarisation compared to M0 (Figure 4C). Functional macrophages in direct or indirect contact with both blank and HSA-alone MPs demonstrated no apparent level of expression of both pro-inflammatory-like (TNF-α) and pro-immunoregulatory-like (TGF-β1 and CCL18) cytokine secretion in comparison to functional macrophages.

**FIGURE 5:**
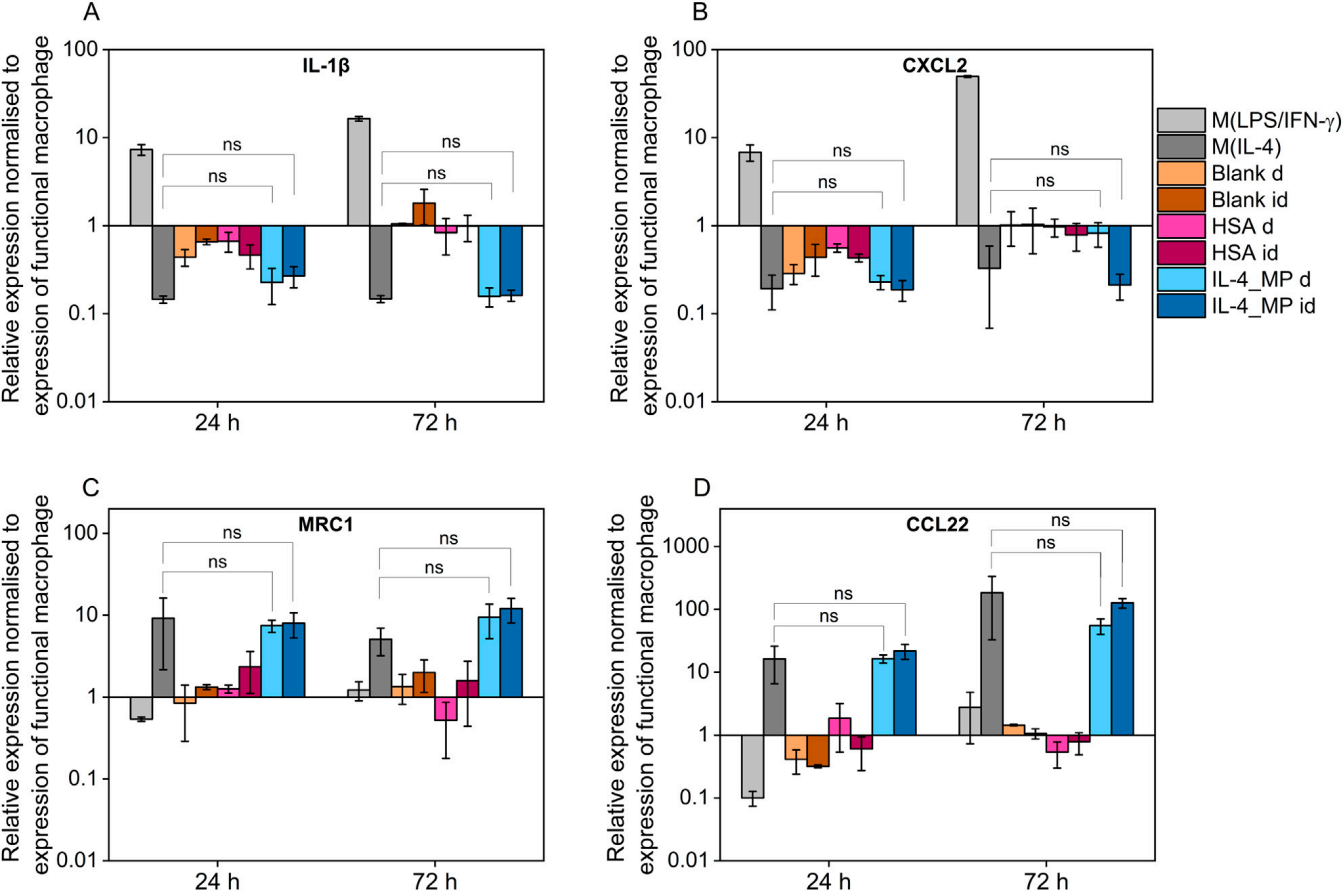
Quantitative PCR assay of **(A)** IL-1β, **(B)** CXCL2, **(C)** MRC1 and **(D)** CCL22 expression after 24 and 72 h exogenous treatments with pro-inflammatory cytokines, pro-immunoregulatory cytokines; blank MPs, HSA-alone MPs, IL-4 encapsulated MPs indirectly through well plate inserts (id), and MPs directly co-cultured with functional macrophages **(D)**. All results were normalised to appropriate GAPDH value, ΔCt, and further normalised to the ΔCt value of the gene of interest of functional macrophages as control, ΔΔCt, using equation 2^−ΔΔCt^. Error bars represent ±standard error; significance determined using one-way ANOVA tests, *: *p* < 0.05, significant; ns: not significant.

THP-1 differentiated macrophages treated with pro-inflammatory factors showed upregulation in expression of both IL-1β and CXCL2 whereas when exposed to IL-4/HSA encapsulated microparticles and M(IL-4), there was a downregulation in expression of IL-1β and CXCL2 (Figures 5A, B). The results were consistent with inflammatory cytokine (TNF-α) expressions measured using an ELISA assay. MRC1 and CCL22 were selected as pro-immunoregulatory markers pro-inflammatory shifted cells (M(LPS/IFN-γ)) showed downregulation in expression of MRC1 and CCL22 whereas upregulation was observed in those of exogenously pro-immunoregulatory shifted M(IL-4) cells and those exposed to IL-4/HSA encapsulated microparticles.

Both blank MPs and HSA-alone MPs either directly or indirectly co-cultured with functional macrophages showed no significant regulatory differences in any markers examined, indicating that MPs and the release of HSA have no effects on macrophage polarisation. IL-4/HSA encapsulated MPs when co-cultured with functional macrophages directly or indirectly separated by a transwell membrane showed downregulation of IL-1β and CXCL2 and upregulation of MRC1 and CCL22 (Figures 5C, D), compared to functional macrophages. There was no significant difference in gene expression when functional macrophages were exposed to IL-4 exogenously or IL-4/HSA microparticles.

## 4 Discussion

Conventional high molecular weight PLGA polymers often provide little control in sustained protein delivery due to long hydrolysis degradation time (Kirby et al., 2011; White et al., 2013). Therefore a low molecular weight hydrophilic PLGA-PEG-PLGA triblock co-polymer (TB) was synthesised and incorporated with PLGA as a release modifier to increase water ingress and facilitate release (Chen et al., 2005). A recent study has shown that microparticles and macrophage co-delivery can be beneficial for angiogenesis (Risser et al., 2023). In this study, we sought to understand the specific kinetics of IL-4 released from PLGA microparticles and the impact on macrophage phenotype *in vitro*. *In vivo* testing of immunomodulatory microparticle systems requires significant use and sacrifice of animals (Hubrecht and Carter, 2019), therefore we sought to develop a suitable *in vitro* pre-screening method of assessing IL-4 functionality in injectable microparticles.

Four commonly used drug release kinetic models were selected to analyse formulation protein release: zero order, first order, the Higuchi and Ritger-Peppas.

Microparticles with IL-4/HSA displayed a 7-day functional cumulative release profile. No apparent release was observed after 7 days for IL-4 in IL-4/HSA encapsulated microparticles (Figure 3). This is potentially due to physical interactions between IL-4 and HSA (Hawe and Friess, 2007). During microparticle fabrication, albumins such as HSA and bovine serum albumin (BSA) have been reported to stabilise cytokines by binding with their hydrophobic regions and preventing aggregation to reduce thermal inactivation (Hawe and Friess, 2007; Finn et al., 2012; Benington et al., 2021). It is possible that the interactions between HSA and IL-4 during microparticle fabrication could alter release kinetics. Functional release of IL-4 best fitted to the first order kinetic model (Figure 3A). This kinetic model describes cumulative drug release mechanism that is concentration dependent (Bruschi, 2015). The amount of drug released decreases with a decreasing concentration gradient over time (Shafiei et al., 2021).

The 28-day functional release of HSA from the IL-4/HSA microparticles best-fitted with the Ritger-Peppas method indicating diffusion controlled release (Figure 3B) (Peppas and Narasimhan, 2014; Su et al., 2023). The encapsulated proteins diffuse from a region of higher concentrations, i.e., water-insoluble PLGA matrices, through to a region of lower concentration, i.e., media. The Higuchi and the Ritger-Peppas method are commonly used to describe drug release behaviour in polymeric systems. However, the Higuchi method may over simplify release and this is often considered unsuitable for use in a three-dimensional release system (Siepmann and Peppas, 2011). The functional release of IL-4 displayed the same release kinetics at 7 days and 28 days whilst HSA release kinetics best fitted to different models for the 7 days release compared to 28 days release (Supplementary Table S1; Supplementary Figure S1). It is worth noting that IL-4 functional release plateaued beyond 7 days with slope of <5 × 10^−4^ for release between day 9 and 28, while HSA continued to release for up to 28 days, therefore HSA release kinetics were fitted to the 28 days release profile (Supplementary Figure S3). For each of the two batches of IL-4/HSA microparticles there was no significant difference in the best-fit release kinetics for IL4 (*p* = 0.058) or HSA (*p* = 0.681). This indicates that release kinetics of microparticles formed from PLGA85:15 with 40% TB are controlled between separate batches.

The differences between IL-4 and HSA functional release kinetics from IL-4/HSA microparticles could be due to the physical characteristic differences such as molecular weight (14.9 and 66.7 kDa, respectively) and isoelectric points (9.71 and 4.7, respectively). In addition, the increased sampling time periods after 7 days could have resulted in some IL-4 degradation in between release and sample collections due to much shorter half-life (approx. 20–30 min) compared to HSA (19 days) (Shintani et al., 2017; Tao et al., 2021).

Primary peripheral-blood monocytes (PBMs) are often used to generate macrophages as an *in vitro* system (Scherr et al., 2023; Sermet et al., 2021; Ziemba et al., 2019). However, due to limited lifespan and biological variabilities of PBMs, leukaemia monocytic cell lines such as THP-1 are often considered as an alternative to primary monocytes due to their proliferative characteristics and similar polarisation response to external stimuli (Chanput et al., 2014). In response to PMA, THP-1 cells differentiate into a macrophage-like phenotype mimicking primary human macrophages (Lund et al., 2016). Following exposure to polarisation stimuli, similar to primary human macrophages, THP-1 differentiated macrophages polarise towards pro-inflammatory (M1)-like and pro-immunoregulatory (M2)-like phenotypes (Sica and Mantovani, 2012; Tedesco et al., 2018). Although THP-1 differentiated macrophages and primary human monocyte differentiated macrophages have distinct developmental origins and phenotypic attributes, it has been suggested that these two distinctive cell types may have similarity in selected functional assays (Tedesco et al., 2018). THP-1 macrophages have been widely used in studies to explore M1-and M2-like macrophage polarisation pathways in response to exogenous treatments (Chanput et al., 2014; Maess et al., 2010; Genin et al., 2015; Abuawad et al., 2020; Pinto et al., 2021).

Exogenous treatments of THP-1 differentiated macrophages were effective in generating M1-and M2-like phenotypes, measured by ELISA and qPCR (Figures 4, 5). Microparticle candidates were applied to differentiated, non-polarised THP-1 macrophages either via transwell inserts or by direct contact. Transwell inserts of 1 µm membrane were used to ensure sufficient delivery of cytokines whilst microparticles remained in the transwell inserts. The combination of these assays allowed us to assess whether the microparticles were inert or induced any inflammation or undesired immunomodulation.

Blank and HSA-alone microparticles were assessed for potential non-targeted effects on macrophage phenotype. Both types of microparticles had no apparent impacts on TNF-α, CCL18 and TGF-β1 secretion nor IL-1β, CXCL2, MRC1 and CCL22 expression compared to non-polarised macrophages. This indicates that polymeric microparticles as drug carriers and HSA as a protein carrier had no effect on macrophage phenotypic modulation. It is worth noting that macrophages secrete low levels of TGF-β1 (Ashcroft, 1999) as observed in the increased cumulative secretion in Figure 4 between 24 and 72 h. The upregulated TGF-β1 secretion is indicative of a repair response in macrophages (Roman-Blas et al., 2007; Liu et al., 2018).

Exogenous stimulation of IL-4 was used to polarise macrophages towards a pro-immunoregulatory-like phenotype, which was confirmed with detectable expression of CCL18 and increased expression of TGF-β1; along with upregulation of immunomodulatory markers, MRC1 and CCL22, and downregulation of inflammatory markers, IL-1β and CXCL2. These findings align with exogenous treatments of macrophages indicated in previous studies (Baxter et al., 2020; Tedesco et al., 2018). Non-polarised macrophage co-culture with IL-4/HSA microparticles either directly or indirectly through transwells displayed detectable expression of CCL18 and increased expression of TGF-β1, comparable to exogenous addition of IL-4. Gene expression of MRC1 and CCL22 in these cells also displayed upregulation with no significant difference to cells exposed to exogenous addition of IL-4.

The results indicate that the encapsulation method of IL-4 within microparticles did not result in any apparent functionality alterations to IL-4; IL-4 encapsulated microparticles had a sustained impact on macrophage phenotype. Furthermore, IL4/HSA release kinetics were controlled between separate batches of microparticles. The THP-1 assay developed in this study demonstrates robust and validated modulation of macrophage phenotype and offers a platform for pre-screening of cytokine and drug release, beyond the work demonstrated herein with IL-4.

## 5 Conclusion

We designed a polymeric microparticle system that showed controlled functional release of IL-4/HSA over 7 days. IL-4 release kinetics fit with the first-order release kinetics model, indicating concentration dependent release. A THP-1 based *in vitro* assay was developed to investigate cell response to IL-4 release *in situ*. Controlled IL-4 release from polymeric microparticles provided a positive immunomodulatory response similar to exogenously IL-4 stimulated cells, The robust, validated THP-1 *in vitro* assay provides an avenue to reduce reliance on *in vivo* testing and may be employed to give valuable insight into immune-modulation of macrophage phenotype when co-cultured with microparticles encapsulated with drugs and cytokines.

## Data Availability

All data created during this research are openly available from the University of Nottingham data repository at http://doi.org/10.17639/nott.7484
