# Supplementary material for: Functional interleukin-4 releasing microparticles impact THP-1 differentiated macrophage phenotype
Source: Front Bioeng Biotechnol. 2024 Nov 5;12:1496111. doi: 10.3389/fbioe.2024.1496111 (PMC11573512; doi:10.3389/fbioe.2024.1496111)
Supplement: Supplementary file 1 [file DataSheet1.docx]

Supplementary Material

# Supplementary Figures and Tables

Table S1. Kinetics parameters of protein released from microparticles for 7 days release.

| Model | Equation | Parameters | IL-4 N1 | IL-4 N2 | HSA N1 | HSA N2 |
| --- | --- | --- | --- | --- | --- | --- |
| Zero order | Q = K_0_t+Q_0_ | K_0_ | 0.019 | 0.020 | 1.555 | 1.268 |
|  |  | Q_0_ | 0.070 | 0.176 | 16.180 | 22.801 |
|  |  | R^2^ | 0.876 | 0.685 | 0.748 | 0.711 |
| First order | Q = 1 - *e*^-K^_1_^t^ | K_1_ | 0.638 | 0.886 | 1.091 | 1.274 |
|  |  | R^2^ | **0.996** | **0.983** | **0.977** | **0.928** |
| Higuchi | Q = K_H_t^1/2^ | K_H_ | 0.080 | 0.136 | 12.109 | 14.154 |
|  |  | R^2^ | 0.918 | 0.364 | -0.324 | -2.377 |
| Ritger-Peppas | Q = Kt^n^ | K | 0.088 | 0.185 | 16.982 | 22.830 |
|  |  | R^2^ | 0.960 | 0.864 | 0.922 | 0.918 |

Q is the cumulative amount of drug released at time t; K_0_, K_1_, K_H_, and K are regarded as kinetic constant for zero-order release, first-order release, Higuchi, and Ritger-Peppas, respectively. and n is the diffusional exponent denoting the drug transport mechanism.


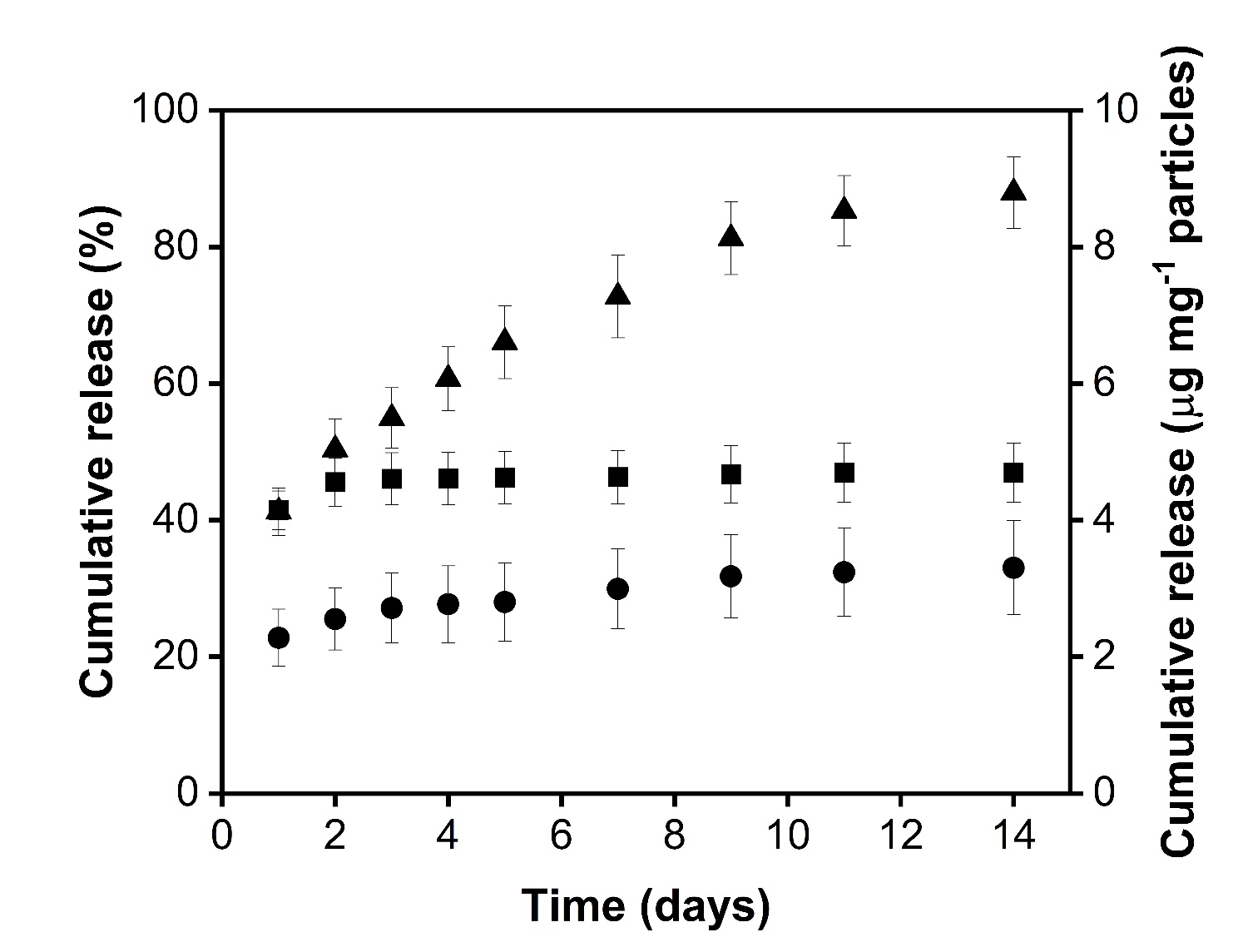


**Figure S1. Cumulative release of microparticles formulated with various TB percentage; 0% (■), 20% (●), and 40% (▲).**


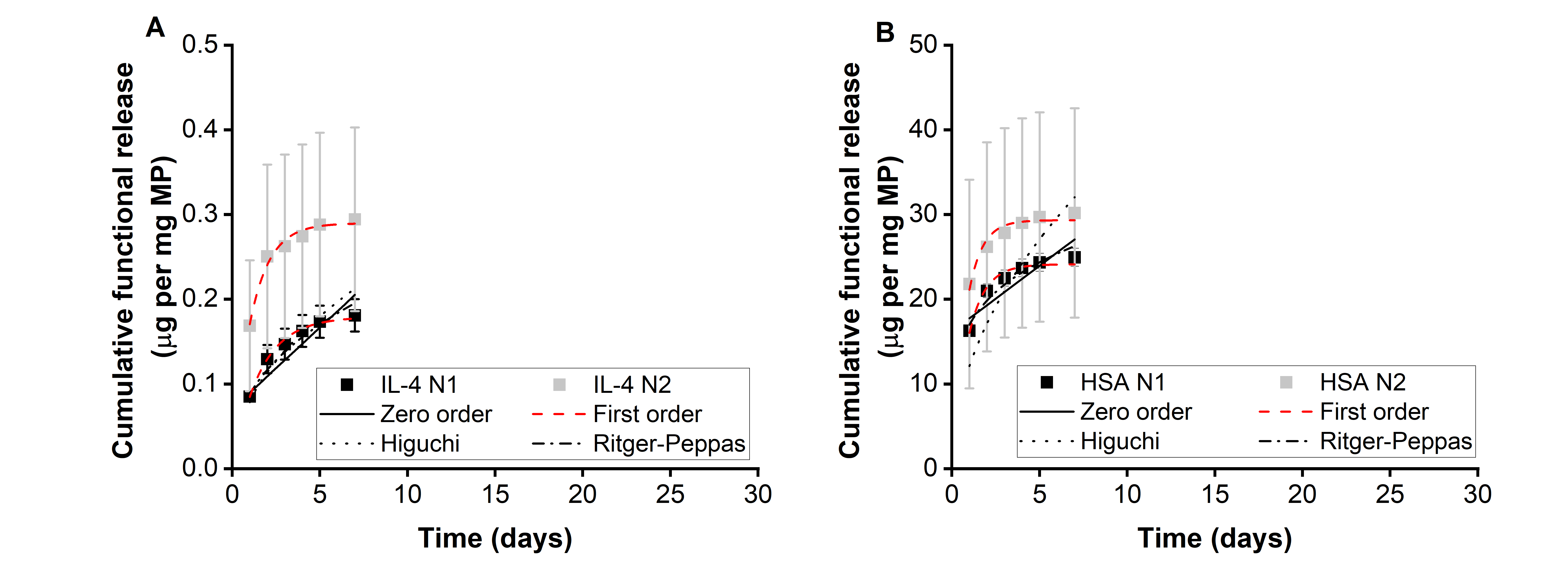


**Figure S2.** Functional protein release of microparticles measured using ELISA. Kinetics models fitted for 7 days of release. Cumulative release of (A) IL-4, encapsulated with (B) HSA, n=3. Error bars represent ± cumulative standard deviation.


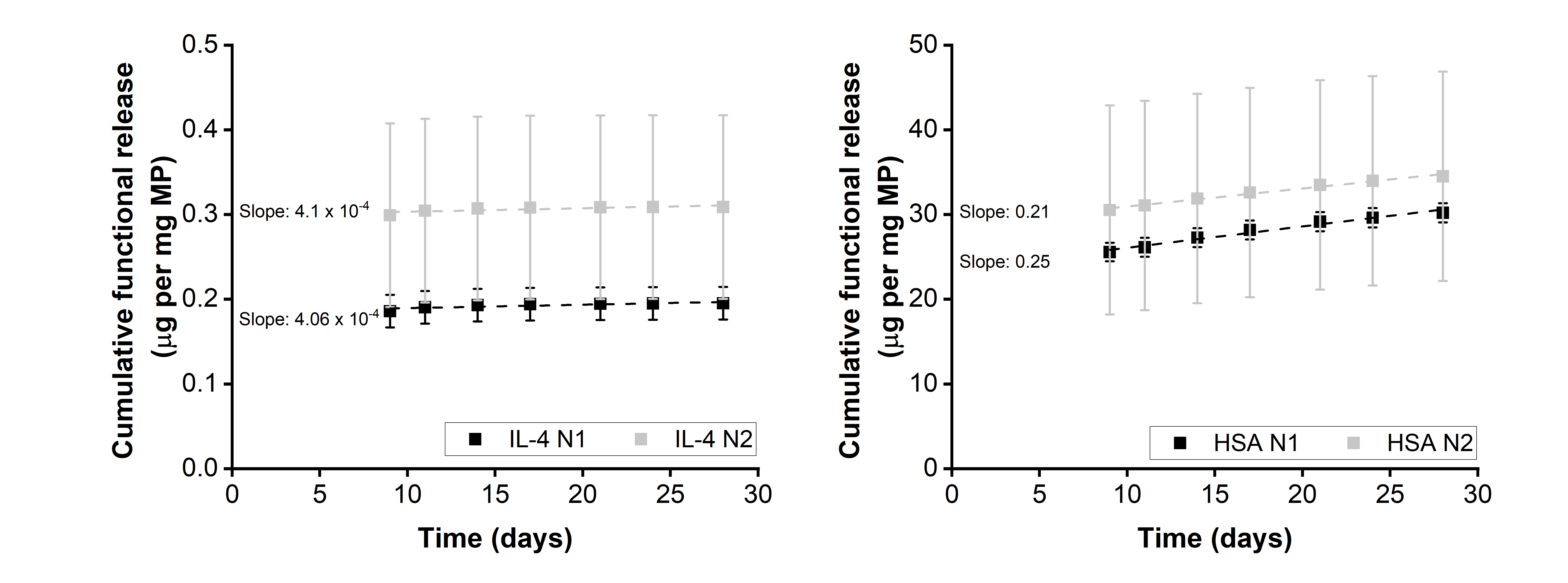


**Figure S3.** Functional protein release of microparticles measured using ELISA. Linear fit for day 9 to 28 of release. Cumulative release of (A) IL-4, encapsulated with (B) HSA, n=3. Error bars represent ± cumulative standard deviation.
